# Lower lung donor PaO_2_/FiO_2_ ratio correlates with early primary graft dysfunction, but does not impact overall survival after lung transplantation: a dual center study

**DOI:** 10.3389/ti.2026.16414

**Published:** 2026-10-09

**Authors:** Rene Novysedlak, Cedric Vanluyten, Jan Van Slambrouck, Annalisa Barbarossa, Hanne Beeckmans, Dirk Van Raemdonck, Paul De Leyn, Hans Van Veer, Lieven Depypere, Yanina Jansen, Bart M. Vanaudenaerde, Robin Vos, Ann Belmans, Andrea Zajacova, Monika Svorcova, Jaromir Vajter, Janis Tavandzis, Jiri Pozniak, Jan Simonek, Zuzana Ozaniak Strizova, Jiri Vachtenheim, Robert Lischke, Laurens J. Ceulemans

**Affiliations:** 1 Department of Thoracic Surgery, University Hospitals Leuven, Leuven, Belgium; 2 Prague Lung Transplant Program, 3rd Department of Surgery, First Faculty of Medicine, Charles University, Motol and Homolka University Hospital, Prague, Czechia; 3 Laboratory of Respiratory Diseases and Thoracic Surgery, BREATHE, Department of CHROMETA, KU Leuven, Leuven, Belgium; 4 Department of Respiratory Diseases, University Hospitals Leuven, Leuven, Belgium; 5 Department of Public Health and Primary Care, Leuven Biostatistics and Statistical Bioinformatics Center (L- BioStat), KU Leuven, Leuven, Belgium; 6 Prague Lung Transplant Program, Department of Pneumology, Second Faculty of Medicine, Charles University, Motol and Homolka University Hospital, Prague, Czechia; 7 Department of Anesthesiology, Resuscitation and Intensive Care Medicine, Second Faculty of Medicine, Charles University, Motol and Homolka University Hospital, Prague, Czechia; 8 Department of Immunology, Second Faculty of Medicine, Charles University, Motol and Homolka University Hospital, Prague, Czechia

**Keywords:** donor PaO_2_/FiO_2_ ratio, extended criteria donor, lung donor, lung transplantation, primary graft dysfunction

## Abstract

Extended criteria donor lungs falling below the arbitrarily set PaO_2_/FiO_2_ (PFR) threshold of 300 are frequently declined despite other acceptable parameters. This two-center retrospective analysis investigates whether donor PFR is a risk factor for outcomes after lung transplantation (LuTx). A retrospective cohort analysis was conducted at University Hospitals Leuven, Belgium and Motol University Hospital, Prague, Czech Republic including adult LuTx recipients from 2010 to 2022 (Leuven) and 2018–3/2023 (Prague). Primary endpoints: primary graft dysfunction (PGD) grade 3 at 24 and 72 h and 2‐year overall survival. Secondary endpoints: incidence of ACR and CLAD-free survival. Donor PFR was measured from an arterial blood gas sample. Logistic and Cox regression analyses were employed to assess correlations, while survival was analyzed using Kaplan-Meier estimates. A total of 984 LuTx recipients were included. Median follow-up was 4 years. Recipients were predominantly male (54%), with chronic obstructive pulmonary disease being the most common indication for transplantation. Donor characteristics included median age of 51 years, and 18% of donors were DCD. Analyses identified lower donor PFR as a risk factor for PGD grade 3 at 24 h, but not at 72 h. PFR was not associated with the incidence of ACR or with 2-year overall survival after LuTx. Donor PFR does not appear to be a risk factor for development of late PGD or for adversely impacting outcomes. However, it is associated with increased risk of early PGD.

## Graphical Abstract

**Figure:**
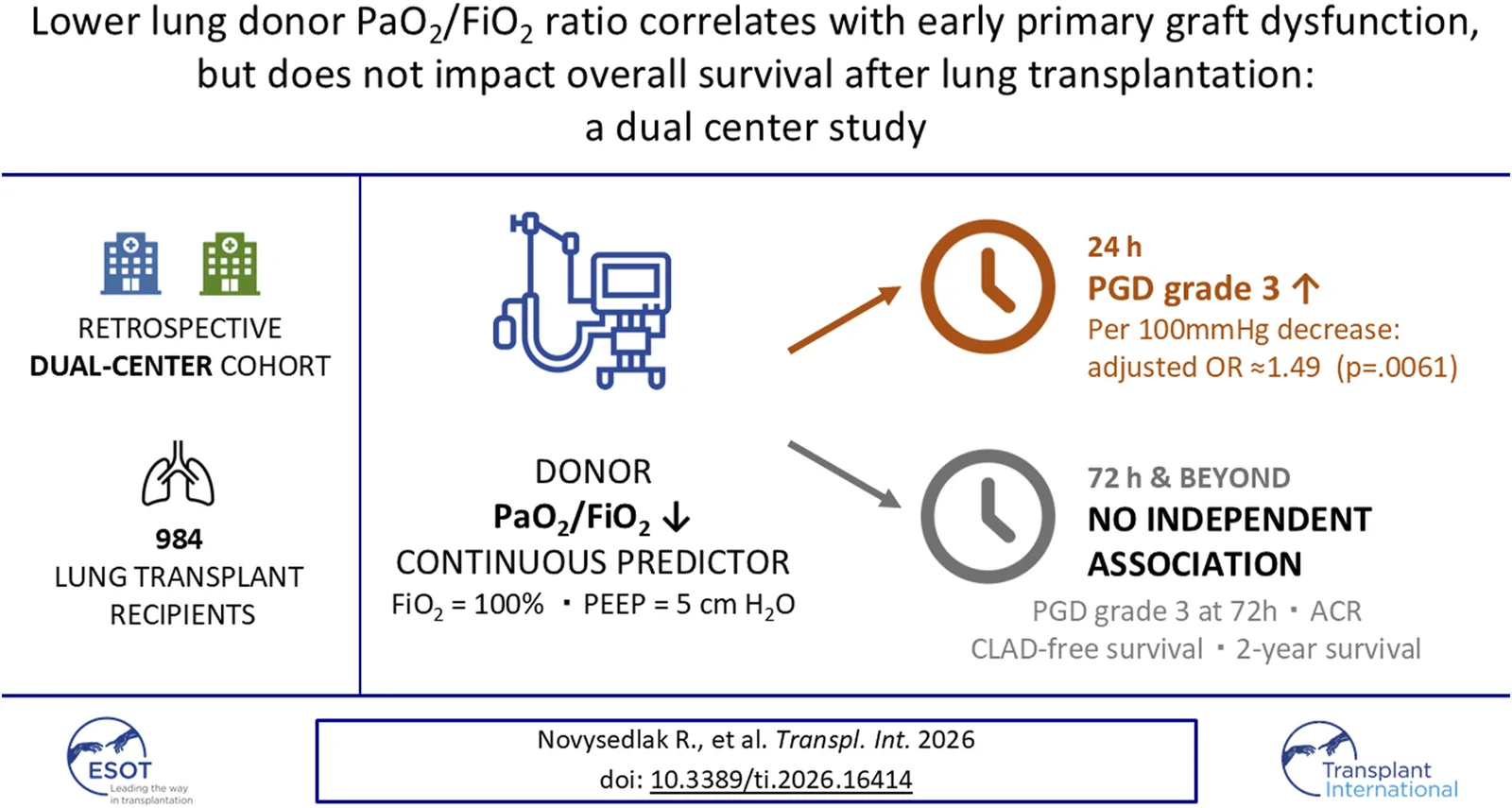

## Introduction

Despite improvements in donor management and organ preservation, the potential of donor lungs remains underutilized. A significant proportion of donor lungs are declined, contributing to waiting list mortality [1]. Increased utilization of extended criteria donor (ECD) lungs could help to bridge the gap between the growing need for transplantation and the limited availability of suitable organs.

ECD lungs that do not meet traditional standards for donor lung selection, are expected to be evaluated with increased caution. However, the definition of what qualifies as “extended” is evolving [2]. A notable illustration of this shift is donation after circulatory death (DCD), which, although still officially classified as an extended criterion, has become so widely adopted in many transplant programs that it is increasingly regarded as part of standard donor practices rather than being truly extended [3]. Among the key parameters historically incorporated into donor lung acceptability criteria, the donor PaO_2_ (arterial oxygen tension)/FiO_2_ (fractional inspired oxygen) ratio (PFR) has been widely used as a surrogate marker of donor lung oxygenation and overall suitability for transplantation, although the clinical relevance of fixed threshold values remains debated [4–6]. Concerns persist regarding lower PFR values and their potential association with adverse post-transplant outcomes [7–9]. However, as early as 2008, our group already argued that a low PFR alone should not constitute an absolute contraindication to donor lung utilization [10].

Although donor PFR is widely used as a simple marker of donor lung oxygenation, its physiological interpretation is complex. A reduced PFR may reflect true impairment of pulmonary gas exchange, including intrapulmonary shunt caused by atelectasis, edema, aspiration, infection, or acute lung injury. However, PFR is also influenced by extrapulmonary factors, particularly mixed venous oxygen saturation, cardiac output, hemoglobin concentration, and the balance between systemic oxygen delivery and consumption [11]. Therefore, a low donor PFR should not automatically be interpreted as irreversible structural lung injury, especially when assessed in unstable donors or under variable hemodynamic conditions. In the context of persistent lung donor shortage, understanding the relationship between donor PFR and recipient outcomes is essential for expanding the donor pool and optimizing organ allocation. Several studies have evaluated various risk factors for primary graft dysfunction (PGD) development, but the prognostic value of pre-procurement PFR on PGD remains underexplored [12–14]. This two-center retrospective analysis aims to investigate whether donor PFR is an independent risk factor for early/late PGD and post-transplant survival.

## Patients and methods

### Study design

A retrospective two-center cohort analysis was performed at the University Hospitals Leuven (UZL, Belgium) and Motol University Hospital in Prague (FNM, Czech Republic). Adult patients (≥18 years old) who underwent LuTx between 01/2010 and 12/2022 (Leuven) and 01/2018–03/2023 (Prague) were included. The final date of follow-up was October 31, 2023. The different inclusion periods reflected center-specific availability of complete and consistently retrievable data for the variables required for this analysis, particularly donor PFR, PGD grading, and post-transplant outcomes. Retransplant patients and combined organ transplant patients were excluded (Figure 1). Patients with missing key data were also excluded. All recipients provided written informed consent to approve use of medical data for research. The study was approved by the research ethics committee UZ/KU Leuven (S68554) and FN Motol (EK-530/21) and adhered to the principles of the Declaration of Helsinki and is compliant with the International Society for Heart and Lung Transplantation (ISHLT) Ethics statement.

**FIGURE 1:**
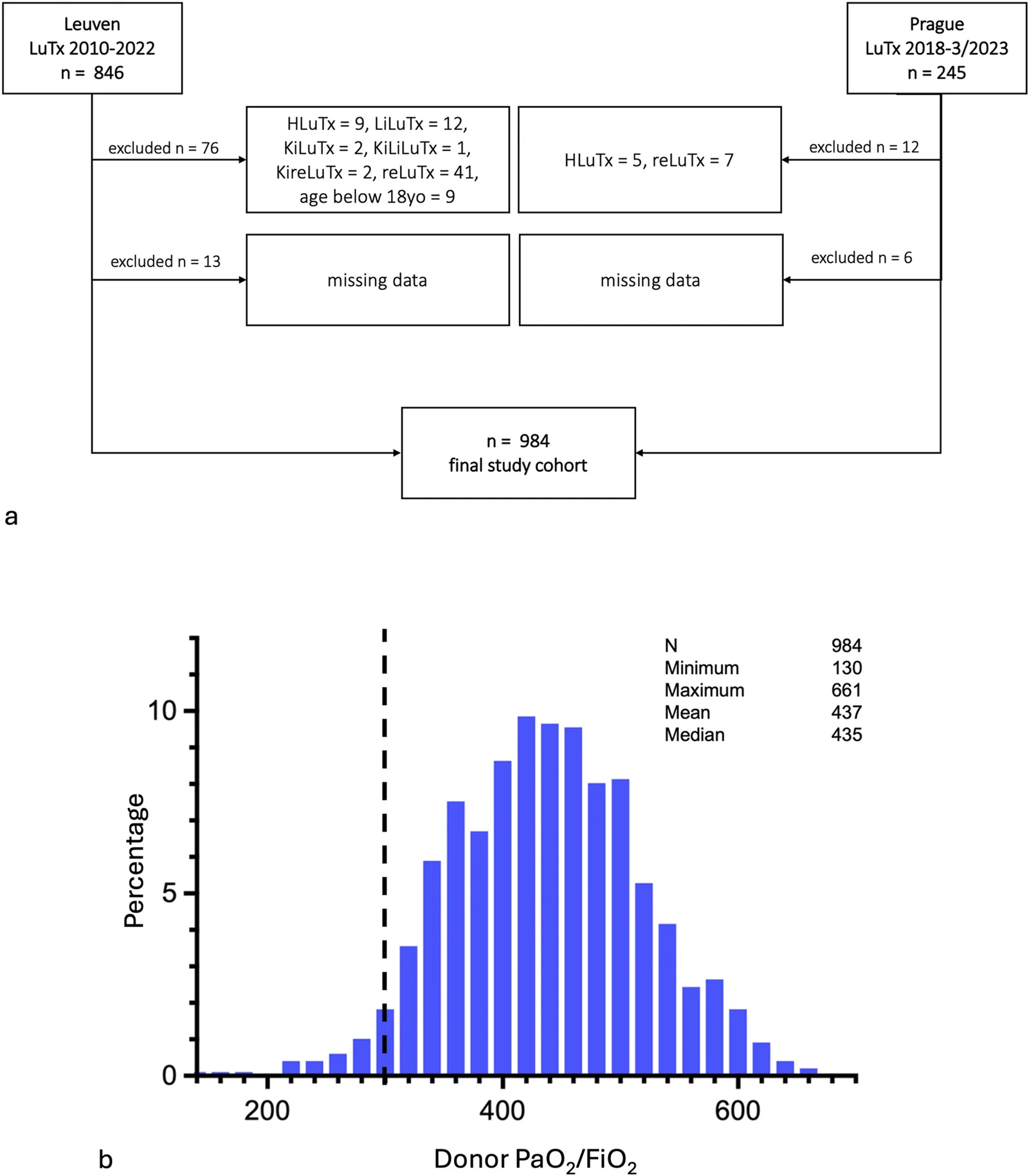
**(a)** Flowchart diagram of the study cohort. Abbreviations: LuTx, lung transplantation; HLuTx, heart-lung transplantation; LiLuTx, liver-lung transplantation; KiLuTx, kidney-lung transplantation; KiLiLuTx, kidney-liver-lung transplantation; KireLuTx, kidney-lung re-transplantation; reLuTx, lung re-transplantation; **(b)** Distribution of last donor PaO_2_/FiO_2_ in the cohort. The y-axis represents the percentage of donors within each PaO_2_/FiO_2_ interval, illustrating the relative frequency distribution of the variable across the donor population (N = 984).

### Study population

Donor and recipient demographics as well as surgical and postoperative parameters were selected based on prior studies [15–20].

Donor and preservation variables included: donor type (donation after brain death [DBD] vs. DCD), age, sex, body mass index (BMI), duration of ventilation, duration of *ex-vivo* lung perfusion (EVLP), total ischemic time of first/second lung and the donor PFR. EVLP was used according to center practice for marginal/uncertain lungs, including impaired oxygenation, DCD/ECD features, radiographic findings, or other clinical concerns. Although donor smoking and heavy alcohol use were associated with an increased risk of PGD [21, 22], they were not included as variables in our analysis due to the large number of missing data.

Recipient variables were: age, sex, BMI, indication for transplantation, type of transplant, surgical approach, time on post-operative ECMO (extracorporeal membrane oxygenation, days), PGD grade at 0, 24, 48 and 72 h, length of intensive care unit (ICU) stay (days), length of hospital stay (days), grade and incidence of acute cellular rejection (ACR) within the first post-transplant year, time to CLAD or death (due 9/2022 for UZL and 9/2023 for FNM) and patient survival. A 2-year survival endpoint was selected to reflect early to intermediate-term outcomes influenced by donor and perioperative factors, while reducing confounding from long-term comorbidities. The immunosuppression protocol followed the profile previously described by Provoost et al. (Chapter 2.4) [23].

### Outcome

Primary endpoints were PGD grade 3 at 24 and at 72 h and 2-year patient survival. Secondary endpoints were the incidence of ACR within the first year after transplantation and CLAD-free survival. PGD was diagnosed based on the ISHLT consensus and was assessed by pulmonary edema on X-ray and the ratio of arterial partial pressure of oxygen to the fraction of inspired oxygen (PaO_2_/FiO_2_) [24]. Data on blood gases were acquired by extraction of electronic patient files. Early PGD was defined as PGD grade 3 at 24 h, late PGD as grade 3 at 72 h. Histological diagnosis of ACR was made using tissue samples obtained from transbronchial biopsy (either by forceps or cryobiopsy), through flexible bronchoscopy. The biopsies underwent evaluation based on ISHLT guidelines [25]. (Supplementary Material 1).

### Donor PFR

Donor PFR was defined as a ratio of arterial oxygen tension (PaO2) to fractional inspired oxygen (FiO2). No *a priori* categorization of donor PFR (e.g., <300 vs. ≥ 300) was used to define comparison groups; donor PFR was modeled as a continuous predictor in all primary analyses. For the Leuven cohort, the PFR was defined as the last value reported by Eurotransplant at the time of organ allocation. For the Prague cohort, it was the last value measured prior to procurement. All donor arterial blood gases were obtained after 10 min of ventilation at an FiO_2_ of 100% and a positive end-expiratory pressure of 5 cm H_2_O, with lung recruitment maneuvers performed beforehand when indicated. Donor PFR in DCD donors was defined using the same center-specific principle as in DBD donors. All donor PFR values were reviewed for plausibility and retained in the analysis if they were documented under the predefined ventilatory conditions and if the lungs were ultimately accepted and transplanted.

### Statistical methods

Statistical analyses were performed using SAS9.4 (Windows) by an experienced biostatistician (ABe). Continuous variables were reported as median (interquartile range), categorical variables as number (percentage). Hospital and ICU duration were summarized using Cumulative Incidence Functions (CIF) to account for the competing risk of in-hospital/ICU death.

Spearman correlations (ρ) and Kruskal–Wallis tests were used to evaluate relations between covariates and PFR.

Associations between P/F ratio and binary outcomes were assessed using logistic regression analyses (PGD>=3, ECMO, Any ACR>0 or 1). Survival outcomes (all-cause death and CLAD-free survival) were analyzed using Cox regression. Competing risk outcomes (ICU and hospital duration) were analyzed by means of a Fine&Gray model. ACR results over time were analyzed using a generalized estimating equations (GEE) multinomial model, while the number of ACR assessment >0/1 was assessed using a multinomial logistic model.

For all of the above models and outcomes, the linearity assumption was assessed for all continuous predictors by checking for improvement in the model when fitting the variables using non-linear functions (log-transformed, quadratic and restricted cubic spline) using Akaike’s Information Criterion (AIC).

The association between PFR and outcome was assessed using univariate models. Multivariable model was used that included all known predictors, to assess whether the association between PFR and outcome was independent from known predictors. As a sensitivity analysis, to assess the stability of the obtained association, a backward model reduction was performed on the known predictors (using p < 0.157 which corresponds to a decrease in AIC) before adding PFR to the model.

Data inspection revealed that the availability of ACR assessments was associated with outcome, i.e., missingness was not at random. Therefore, ACR data are analyzed similar to a pattern-mixture model whereby the number of assessments was added as a factor to the model [26].

All tests were 2-sided and assessed at a significance level of 5%. Due to the retrospective and exploratory nature of the study, no formal adjustment for multiple comparisons was applied; secondary analyses were interpreted descriptively and considered hypothesis-generating.

## Results

### Study population

A total of 846 LuTx procedures were performed in Leuven between January 2010 and December 2022. In Prague, 245 LuTx between January 2018 and March 2023. After exclusion of 88 ineligible procedures, 1,003 eligible double or single LuTx remained. In 19 cases, important data were missing, resulting in 984 patients included in the analysis (Figure 1A). Median follow-up was 4 (1.6–7.4) years.

### Recipient and donor demographics

Recipient and donor demographics are summarized in Table 1. Recipient age was 57 (49–62) years of which 531 (54%) were male and 453 (46%) female. The most common indication for transplantation was chronic obstructive pulmonary disease, accounting for 492 (50%) cases, followed by pulmonary fibrosis (n = 299 [30%]), and cystic fibrosis (n = 106 [11%]). Recipient BMI was 23 (20–27) kg/m2.

**TABLE 1:** Overview of recipient and donor demographics.

Characteristics	Results (n = 984)
Donor	
*Type donor, n (%)*	
DBD	803 (81.60)
DCD	181 (18.39)
Age at donation, years	51 (39–61)
*Sex, n (%)*	
Male	541 (54.98)
Female	443 (45.02)
Body mass index, kg/m^2^	24 (22–27)
Time on ventilator, days	3 (2–6)
Last PaO_2_/FiO_2_, mmHg	435 (380–492)
Recipient	
Age at transplant, years	57 (49–62)
*Sex, n (%)*	
Male	531 (53.96)
Female	453 (46.04)
Body mass index, kg/m^2^	23 (20–27)
*Indication for transplant, n (%)*	
ILD	299 (30.39)
CF	106 (10.77)
COPD	492 (50)
PAH	47 (4.78)
Other	40 (4.06)
Pre-operative ECMO support	36 (3.65)
Surgical	
EVLP, n (%)	26 (2.64)
EVLP duration, min	250 (163.5–316)
*Type of transplant, n (%)*	
Double lung	973 (98.87)
Single lung	11 (1.12)
*Type of incision, n (%)*	
Thoracotomy (bilateral and unilateral)	722 (73.52)
Clamshell	260 (26.48)
*Total ischemic time, min*	
First lung	226 (182–275)
Second lung	382 (325–447)
Intra-operative ECLS	312 (31.70)

Data are expressed as median (IQR) if not otherwise indicated. Data on incision type were available for 982 recipients. Abbreviations: CF, cystic fibrosis; COPD, chronic obstructive pulmonary disease; DBD, donor after brain death; DCD, donor after circulatory death; ECMO, extracorporeal membrane oxygenation; EVLP, *ex-vivo* lung perfusion; ILD, interstitial lung disease; PAH, primary arterial hypertension.

Donors were aged 51 (39–61) years, with a BMI of 24 (22–27) kg/m^2^. The donor population comprised 541 (55%) male and 443 (45%) female donors. DCD procurement was performed in 181 (18%) cases. In the present cohort, only three (1.6% of DCD) DCD donors were classified as Maastricht category IV, while all remaining DCD donors were category III [27]. Median time on ventilator was 3 (2–6) days. Median donor PFR was 435 (380–492). The distribution of PFR in 20-mmHg intervals is illustrated in Figure 1B. Supplementary Figure S1 shows the median and range (maximum and minimum) of PFR by year across the study period. No donor was supported with VA-ECMO at the time of donor PFR assessment.

Median total ischemic time for the first and second implanted lung was 226 (182–275) min and 382 (325–447) min, respectively. EVLP was performed in 26 (3%) cases and lasted 250 (164–316) min. Among donors managed with EVLP in the present cohort, donor PFR values ranged from 311 to 561 mmHg. Therefore, EVLP was not triggered exclusively by a PFR threshold below 300 mmHg, but was used as part of a broader clinical evaluation of donor lung suitability. Double LuTx was performed in 973 (99%) and single LuTx in 11 (1%) cases.

One hundred thirty-nine (14%) LuTx recipients developed PGD 3 at 24 h and 115 (12%) had PGD 3 at 72 h. Two hundred seventy-eight (29%) patients developed PGD 3 within the first 72 h. Transplant outcomes are summarized in Table 2. ICU stay was 8 (5–16) days. Post-operative ECMO was required in 79 (8%) patients and lasted 4 (2–6) days. At least one episode of ACR in A grade was observed in 293/843 (35%) cases. The total length of in-hospital stay was 28 (22–41) days. One, 2-, and 5- year patient survival was 89%, 84%, and 75%, and incidence of CLAD or death was 10%, 19%, and 36%, respectively. Because of differences in inclusion period and perioperative practice between centers, center-specific descriptive data are provided in Supplementary Table S1.

**TABLE 2:** Overview of post-operative outcomes after lung transplantation.

Outcomes	Results
PGD grade 3 at 0h, n (%)	176 (18.62)
PGD grade 3 at 24h, n (%)	139 (14.37)
PGD grade 3 at 48h, n (%)	130 (13.44)
PGD grade 3 at 72h, n (%)	115 (12.02)
Any PGD grade 3 ≤ 72h, n (%)	278 (29.48)
Post-operative ECLS, n (%)	79 (8.03)
Time on post-operative ECLS, days	4 (2–6)
ICU length of stay, days	8 (5–16)
Total hospital length of stay, days	28 (22–41)
ACR grade A ≥ 1, n/N (%)	293/843 (34.76%)
Survival	
Overall patient survival, years since transplant, % (95% CI)	-
1 year	89.4 (87.3–91.2)
2 years	84 (81.5–86.2)
5 years	75.2 (72.1–78.1)
Time to CLAD or death, years since transplant, % (95% CI)	-
1 year	9.9 (8–12.1)
2 years	19.2 (16.6–22.1)
5 years	35.8 (32.3–39.5)

Data are expressed as median (IQR) if not otherwise indicated. Percentages for PGD outcomes were calculated based on available outcome data: n = 945 at 0 h, n = 967 at 24 h, n = 967 at 48 h, n = 957 at 72 h, and n = 943 for the derived endpoint of any PGD grade 3 within 72 h.

Abbreviations: ACR, acute cellular rejection; CI, confidence interval; CLAD, chronic lung allograft dysfunction; ECLS, extracorporeal life support; ICU, intensive care unit; PGD, primary graft dysfunction.

### Potential confounding factors for PFR

Relations between potential confounders and PFR were explored (Table 3). A negative correlation was observed between PFR and increasing age of both recipient and donor (ρ = −0.0786, p = 0.0136 and ρ = −0.0871, p = 0.0062), as well as increasing BMI of the recipient and donor (ρ = −0.0638, p = 0.0453, and ρ = −0.2634, p<0.0001) and longer time on ventilator (ρ = −0.0852, p = 0.0075). Lower PFR was observed when recipient was male (p = 0.0469).

**TABLE 3:** Baseline characteristics associated with donor PaO_2_/FiO_2_.

Continuous variables	Spearman Rho	p value
Donor age, years	−0.0871	**0.0062**
Donor BMI, kg/m^2^	−0.2634	**<0.0001**
Time on ventilator, days	−0.0852	**0.0075**
Total ischemic time second lung, min	0.0184	0.5674
Recipient age, years	−0.0786	**0.0136**
Recipient BMI, kg/m^2^	−0.0638	**0.0453**

Categorical variables	PaO_2_/FiO_2_	p value
Donor sex	​	0.1037
Male	433 (373–490)	​
Female	439 (389–497)	​
Donor type: DCD	​	0.1012
No	437 (382–494)	​
Yes	428 (367–483)	​
Preservation type: EVLP	​	0.8547
No	435 (381–491)	​
Yes	435 (375–492)	​
Recipient sex	​	**0.0469**
Male	432 (373–489)	​
Female	441 (389–501)	​
Recipient indication for transplantation	​	0.0739
ILD	427 (372–486)	​
CF	449 (404–513)	​
COPD	436 (382–489)	​
PAH	442 (397–509)	​
Other	452 (371–504)	​

Data are expressed as median (IQR). Bold values indicate statistically significant results (P < 0.05).

These analyses were descriptive and do not imply causal relationships between recipient variables and donor PFR.

Abbreviations: BMI, body mass index; DCD, donor after circulatory death; DBD, donor after brain death; EVLP, *ex-vivo* lung perfusion; ILD, interstitial lung disease; CF, cystic fibrosis; COPD, chronic obstructive pulmonary disease; PAH, primary arterial hypertension.

### PFR is an independent predictor for development of PGD 3 at 24 h

#### Univariable analysis

Univariable analysis demonstrated a trend toward statistical significance (OR 0.998, 95% CI [0.996–1.000] p = 0.0535) for lower PFR being correlated with the development of PGD 3 at 24 h (Table 4).

**TABLE 4:** Univariable and multivariable logistic regression for PGD grade 3 at 24 h and 72 h.

​	At 24 h				At 72 h			
​	Univariable logistic regression		Multivariable logistic regression		Univariable logistic regression		Multivariable logistic regression	
Variables	OR (95% CI)	p value	OR (95% CI)	p value	OR (95% CI)	p value	OR (95% CI)	p value
Donor								
*Type donor*								
DCD	1.131 (0.721–1.775)	0.5923	1.494 (0.856–2.609)	0.1580	0.975 (0.589–1.613)	0.9212	1.139 (0.618–2.097)	0.6768
DBD	#	​	#	​	#	​	#	​
Donor age, years	0.999 (0.988–1.010)	0.8496	1.007 (0.993–1.021)	0.3172	1.006 (0.994–1.018)	0.3448	1.020 (1.005–1.036)	**0.0093**
*Donor sex*								
Female	0.977 (0.681–1.403)	0.9015	0.768 (0.450–1.312)	0.3343	1.273 (0.862–1.881)	0.2247	0.979 (0.560–1.712)	0.9403
Male	#	​	#	​	#	​	#	​
Donor BMI, kg/m^2^	0.980 (0.939–1.023)	0.3637	0.965 (0.915–1.017)	0.1806	0.943 (0.896–0.993)	**0.0247**	0.919 (0.862–0.978)	**0.0084**
Time on ventilator, days	0.960 (0.914–1.007)	0.0953	0.929 (0.876–0.986)	**0.0146**	0.968 (0.921–1.018)	0.2081	0.959 (0.906–1.015)	0.1451
Donor PaO_2_/FiO_2_	0.998 (0.996–1.000)	0.0535	0.996 (0.994–0.999)	**0.0061**	1.000 (0.997–1.002)	0.8411	0.999 (0.996–1.001)	0.2918
Recipient								
Recipient age at transplant, years	1.092 (0.995–1.199)	**0.0006**	1.074 (0.962–1.200)	0.0595	0.364 (0.216–0.613)	**0.0001**	0.301 (0.117–0.775)	**0.0128**
*Recipient sex*								
Female	1.146 (0.800–1.642)	0.4584	1.442 (0.841–2.473)	0.1836	1.275 (0.863–1.883)	0.2224	1.203 (0.682–2.123)	0.5224
Male	#	​	#	​	#	​	#	​
Recipient BMI, kg/m^2^	1.080 (1.036–1.125)	**0.0003**	1.075 (1.020–1.133)	**0.0069**	1.070 (1.023–1.119)	**0.0032**	1.082 (1.023–1.144)	**0.0058**
*Recipient indication for transplantation*								
ILD	1.074 (0.451–2.557)	**<0.0001**	1.037 (0.392–2.744)	**<0.0001**	1.616 (0.548–4.768)	**0.0001**	1.947 (0.594–6.382)	**<0.0001**
CF	0.393 (0.132–1.167)	​	0.452 (0.143–1.426)	​	1.076 (0.322–3.600)	​	1.095 (0.310–3.870)	​
COPD	0.432 (0.180–1.038)	​	0.633 (0.227–1.764)	​	0.660 (0.222–1.967)	​	1.106 (0.322–3.800)	​
PAH	9.114 (3.265–25.44)	​	9.223 (3.069–27.72)	​	10.89 (3.286–36.11)	​	11.86 (3.339–42.14)	​
Other	#	​	#	​	#	​	#	​
*Recipient incision type*								
Clamshell	2.179 (1.500–3.166)	**<0.0001**	1.305 (0.805–2.114)	0.2805	1.795 (1.192–2.701)	**0.0050**	1.105 (0.658–1.855)	0.7063
Thoracotomy (bilateral and unilateral)	#	​	#	​	#	​	#	​
Total ischemic time second lung, min	​	**0.0005**	​	**0.0068**	​	**0.0044**	​	**0.0367**
Linear term	0.990 (0.982–0.998)	​	0.990 (0.981–1.000)	​	1.003 (1.001–1.004)	​	1.002 (1.000–1.004)	​
RCS term 1	1.067 (1.024–1.112)	​	1.065 (1.017–1.116)	​	​	​	​	​
RCS term 2	0.844 (0.757–0.941)	​	0.847 (0.749–0.958)	​	​	​	​	​
*EVLP*								
Yes	1.084 (0.368–3.195)	0.8835	0.885 (0.243–3.223)	0.8526	0.953 (0.282–3.226)	0.9389	0.992 (0.245–4.022)	0.9913
No	#	​	#	​	#	​	#	​

For donor PaO_2_/FiO_2_, the odds ratio is expressed per 1 mmHg increase. For clinical interpretation, an OR of 0.996 per 1 mmHg increase corresponds approximately to an OR of 1.22 per 50 mmHg decrease and 1.49 per 100 mmHg decrease.

# = reference category for calculation of odds ratio. For continuous variables modeled linearly, the odds ratio reflects the change in odds per 1-unit increase. Recipient age was modeled using a quadratic function at 24 h and a log-transformed function at 72 h. At 24 h, the reported OR corresponds to the linear component of the quadratic model, whereas the p-value reflects the overall association of recipient age with the outcome. Bold values indicate statistically significant results (P < 0.05). Abbreviations: BMI, body mass index; CF, cystic fibrosis; CI, confidence interval; COPD, chronic obstructive pulmonary disease; DBD, donor after brain death; DCD, donor after circulatory death; EVLP, *ex-vivo* lung perfusion; ILD, interstitial lung disease; OR, odds ratio; PAH, primary arterial hypertension; RCS, restricted cubic spline.

#### Multivariable analysis

In multivariable logistic regression model, using the no-model reduction strategy, the negative linear relation between last donor PFR and PGD 3 at 24 h was observed (OR 0.996, 95% CI [0.994–0.999], p = 0.0061) (Table 4). Other variables that proved to be independently associated with PGD 3 at 24 h were donor time on ventilator (OR 0.929, 95% CI [0.876–0.986], p = 0.0146), recipient BMI (OR 1.075, 95% CI [1.020–1.133], p = 0.0069), indication for transplantation (p < 0.0001) and total ischemic time of second implanted lung (p = 0.0068). Similar associations were observed when backward model building was used (Supplementary Table S2; Figure 2A).

**FIGURE 2:**
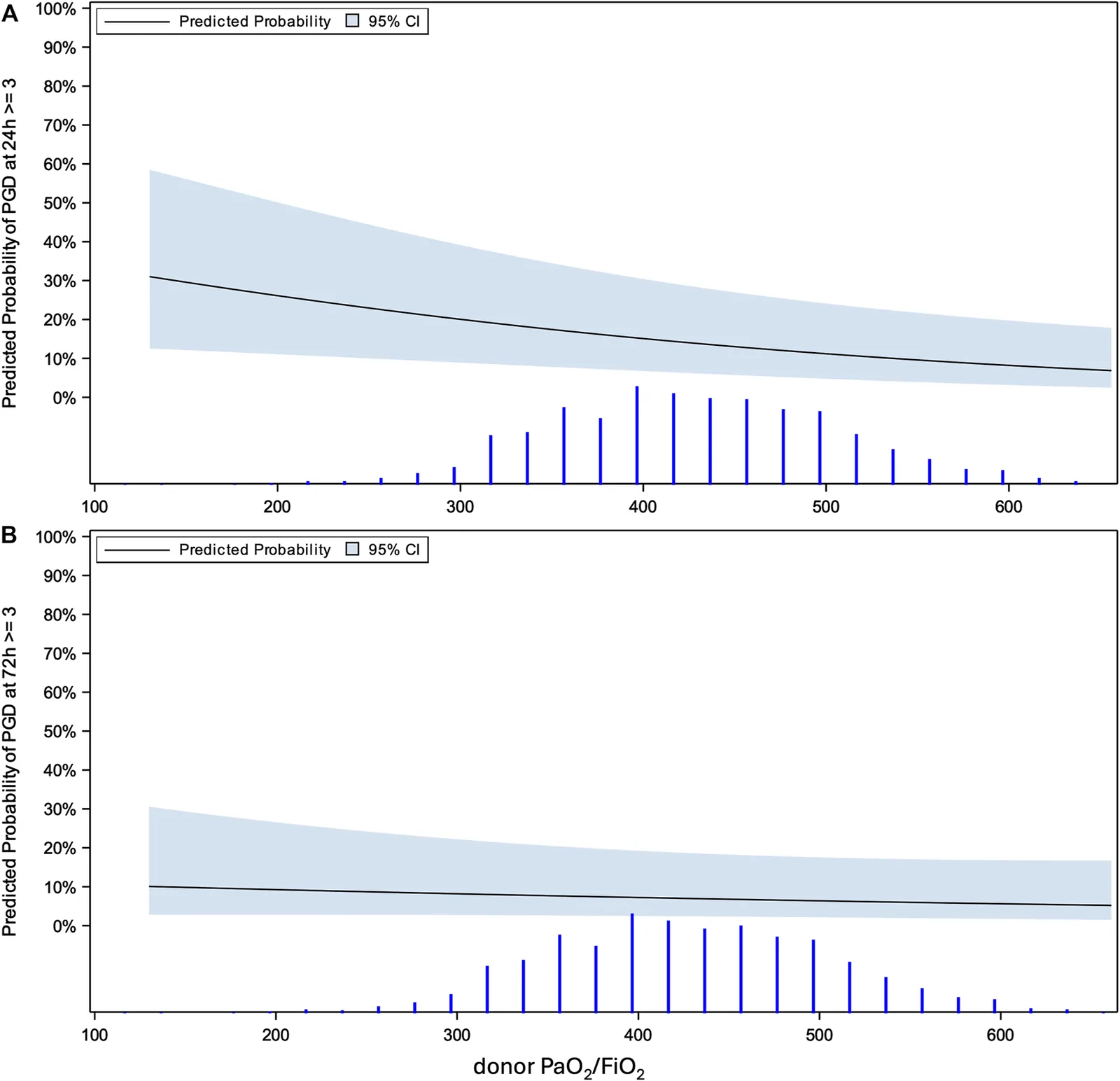
The association between the last donor PaO_2_/FiO_2_ and the predicted probability of PGD grade 3 at **(A)** 24 h and **(B)** 72 h.

Although the per-unit effect size was small, the association becomes more clinically interpretable when expressed across larger PFR differences. Assuming linearity of the fitted model, the observed odds ratio of 0.996 per 1 mmHg increase corresponds approximately to a 1.22-fold increase in the odds of PGD grade 3 at 24 h for each 50 mmHg decrease in donor PFR, and a 1.49-fold increase for each 100 mmHg decrease.

### PFR is not an independent predictor for development of PGD 3 at 72 h

#### Univariable analysis

Univariable analysis did not reveal a statistically significant correlation of PFR with development of PGD 3 at 72 h (Table 4).

#### Multivariable analysis

In multivariable logistic regression model, using the no-model reduction strategy, no significant correlation of PFR with the development of PGD 3 at 72 h was observed (Table 4). Notably, donor age (OR 1.020, 95% CI [1.005–1.036], p = 0.0093) proved to be an independent risk factor for developing PGD 3 at 72 h. Other variables that proved to be independently associated with PGD 3 at 72 h were donor BMI (OR 0.919, 95% CI [0.862–0.978], p = 0.0084), recipient age (OR 0.301, 95% CI [0.117–0.775], p = 0.0128), indication for transplantation (p < 0.0001) and total ischemic time of second implanted lung (OR 1.002, 95% CI [1.000–1.004], p = 0.0367). Similar associations between variables and PGD 3 at 72 h were confirmed when backward model building was used (Supplementary Table S3). The probability for PGD 3 was not associated with decreasing PFR, as illustrated in Figure 2B.

### PFR was not independently associated with the incidence of ACR

Several statistical approaches were employed to reveal potential association of last donor PFR and severity and frequency of ACR grade A in LuTx recipients. No independent association was identified when comparing PFR with ACR outcome, with at least 1 ACR grade A > 0, at least 1 ACR grade A > 0 for patients alive at 6 months, with at least 1 ACR grade A > 1 or number of ACR grade A > 0 or >1 (Table 5).

**TABLE 5:** Univariable and multivariable logistic regression results for the association between PaO2/FiO2 and ACR grade A within 1^st^ year after lung transplantation.

​	Univariable logistic regression		Multivariable logistic regression (no model building)	
Statistical approach to ACR grade A	OR (95% CI)	p value	OR (95% CI)	p value
Any ACR	​	**0.0219**	​	0.4180
*Linear term*	1.006 (1.001–1.012)	​	1.003 (0.997–1.008)	​
*RCS term 1*	0.979 (0.962–0.996)	​	0.990 (0.972–1.008)	​
*RCS term 2*	1.068 (1.002–1.139)	​	1.032 (0.965–1.103)	​
Number of ACR	​	**0.0436**	​	0.1068
*Linear term*	1.007 (1.000–1.013)	​	1.004 (0.997–1.011)	​
*RCS term 1*	0.977 (0.958–0.995)	​	0.983 (0.962–1.005)	​
*RCS term 2*	1.083 (1.010–1.161)	​	1.054 (0.974–1.140)	​

# = reference category for calculation of common odds ratio. For continuous variables, the odds ratio reflects the change in odds when the variable increases by 1 unit. Bold values indicate statistically significant results (P < 0.05). Abbreviations: ACR, acute cellular rejection; CI, confidence interval; OR, odds ratio; RCS, restricted cubic spline.

### Lower PFR has no impact on 2-year survival and CLAD-free survival

Neither univariable (HR 0.999, 95% CI [0.997–1.000], p = 0.1065) nor multivariable (HR 1.000, 95% CI [0.998–1.001], p = 0.5404) analysis revealed a statistically significant association between PFR and overall survival at 2 years post-LuTx. Several correlations of overall survival with other variables were observed (Table 6). Especially, donor and recipient age were significant in both univariable (p = 0.0003 and p < 0.0001) and multivariable analysis (p = 0.0341 and p < 0.0001). Similarly, CLAD-free survival did not demonstrate a significant correlation with PFR (Supplementary Table S4).

**TABLE 6:** Univariable and multivariable Cox regression for all-cause mortality at 2 years.

​	Univariable Cox regression		Multivariable Cox regression	
Variables	HR (95% CI)	p value	HR (95% CI)	p value
Donor				
*Type donor*				
DCD	1.021 (0.758–1.375)	0.8910	0.874 (0.621–1.228)	0.4363
DBD	#	​	#	​
Donor age, years	1.014 (1.006–1.022)	**0.0003**	1.010 (1.001–1.019)	**0.0341**
*Donor sex*				
Female	0.870 (0.690–1.097)	0.2399	0.923 (0.644–1.323)	0.6634
Male	#	​	#	​
Donor BMI, kg/m^2^	​	0.2087	​	0.5541
Linear term	1.196 (0.978–1.461)	​	1.060 (0.870–1.292)	​
Quadratic term	0.997 (0.993–1.001)	​	0.999 (0.995–1.002)	​
Time on ventilator, days	0.993 (0.967–1.020)	0.6255	0.992 (0.963–1.022)	0.5904
Donor PaO_2_/FiO_2_	0.999 (0.997–1.000)	0.1065	1.000 (0.998–1.001)	0.5404
Recipient				
Recipient age at transplant, years	​	**<0.0001**	​	**<0.0001**
Linear term	0.919 (0.860–0.981)	​	0.914 (0.851–0.981)	​
Quadratic term	1.001 (1.001–1.002)	​	1.001 (1.001–1.002)	​
*Recipient sex*				
Female	0.810 (0.642–1.021)	0.0749	0.950 (0.662–1.362)	0.7803
Male	#	​	#	​
Recipient BMI, kg/m^2^	1.045 (1.018–1.072)	**0.0009**	1.011 (0.981–1.043)	0.4685
*Recipient indication for transplantation*				
ILD	1.471 (0.788–2.743)	**0.0011**	1.100 (0.573–2.110)	0.1476
CF	0.534 (0.254–1.122)	​	0.754 (0.341–1.670)	​
COPD	1.068 (0.579–1.970)	​	0.758 (0.393–1.460)	​
PAH	0.996 (0.432–2.299)	​	1.163 (0.491–2.752)	​
Other	#	​	#	​
*Recipient Incision Type*				
Clamshell	1.113 (0.840–1.474)	0.4573	1.057 (0.773–1.446)	0.7272
Thoracotomy (bilateral and unilateral)	#	​	#	​
Total ischemic time second lung, min	1.001 (1.000–1.003)	**0.0235**	1.001 (1.000–1.003)	0.0718
*EVLP*				
Yes	0.796 (0.409–1.548)	0.5015	0.905 (0.455–1.798)	0.7748
No	#	​	#	​

# = reference category for calculation of the hazard ratio. For continuous variables modeled linearly, the hazard ratio reflects the change in hazard per 1-unit increase. Bold values indicate statistically significant results (P < 0.05). Abbreviations: BMI, body mass index; CF, cystic fibrosis; CI, confidence interval; COPD, chronic obstructive pulmonary disease; DBD, donor after brain death; DCD, donor after circulatory death; EVLP, *ex-vivo* lung perfusion; HR, hazard ratio; ILD, interstitial lung disease; PAH, primary arterial hypertension.

## Discussion

The established threshold for PFR in lung donors has historically been arbitrarily set at 300, with donors failing to meet this criterion classified as ECD. While PFR is a widely used indicator of donor lung function, it primarily reflects the efficiency of gas exchange across the blood-gas barrier (BGB). Functional integrity of BGB can be impaired by a wide range of conditions. In the context of lung donation with no known underlying chronic pulmonary disease, common reasons resulting in BGB damage include inflammation, infection, ventilator-induced lung injury and acute lung injury caused by aspiration [28–31]. Lung function is often impaired by the development of atelectasis, resulting in ventilation-perfusion mismatch, which is particularly common in donors with a high BMI [32]. According to Okamoto et al., in these cases, lung recruitment or EVLP allows successful transplantation with good outcomes [32]. Therefore, the evaluation of atelectasis is crucial during donor assessment. When available, computed tomography (CT) imaging should be carefully reviewed. Any areas of atelectasis should be recruited during the macroscopic examination, following bronchoscopy and prior to arterial blood gas sampling.

In DBD donors, all assessments and reinflation of atelectasis are completed antemortem, allowing for lung acceptance prior to the crossclamp. In contrast, for DCD donors, atelectasis is reinflated post-cardiac arrest and during cold flush, with final evaluation on the back table or using *ex vivo* lung perfusion (EVLP) [33]. In many centers, PFR below 300 in DCD also serves as a criterion for utilizing EVLP [34]. In our cohort, fewer than 3% of donor lungs were evaluated using EVLP, making it difficult to extrapolate. However, the true necessity for this procedure has not been fully confirmed [34]. Further studies are needed to determine whether values below 300 in DCD donors truly warrant EVLP, particularly when no therapeutic interventions are applied during the EVLP process. Moreover, several studies have demonstrated that lung transplantation from ECD does not necessarily impair outcomes [4, 35–38]. Strict application of the PFR cutoff may result in unnecessary EVLP or even complete rejection of viable donor lungs [6].

In donors with PFR <300, transplant teams should evaluate whether impaired oxygenation is likely to represent irreversible lung injury or a potentially reversible/extrapulmonary phenomenon. In cases of doubt, a sample can be obtained directly from the pulmonary vein [39]. This also allows for the differentiation of function between individual lobes [40]. In the study by Botha et al., the number of pulmonary veins with PFR below 300 significantly correlated with the incidence of severe PGD [41].

Whitford et al. found no differences in extubation time, PGD, pulmonary function at 6 and 12 months, or 12-month survival between recipients with donor lungs having ICU PFR <300 versus ≥300 [6]. In our study, PGD at 24 h was influenced by donor PFR. No significant impact was observed at 72 h. This supports a potential pathophysiological distinction between early (more inflammatory and hydrostatically driven) and late phenotype of PGD, as previously described by Van Slambrouck et al. [42] Early PGD is likely more influenced by donor-related factors, whereas the development of late PGD appears to be predominantly driven by recipient-specific characteristics. However, this interpretation should be considered hypothesis-generating. Recruitment maneuvers to re-expand atelectatic segments, particularly when combining aggressive manual and ventilator-driven techniques, may cause shear stress, capillary leak, and surfactant depletion. This recruitment-induced lung injury may contribute to the development of early PGD.

However, in the study by Martinsson et al., which examined donor lung weight as a predictor of PGD, donor PFR did not significantly differ between low-weight and high-weight lungs. This finding suggests that PFR may not be a reliable indicator of lung capillary injury [43].

Another notable finding was that donor age was associated with increased odds of PGD grade 3 at 72 h, whereas recipient age showed an inverse association. Previous studies have justified the use of donors over 65 or 70 years, considering both short- and long-term outcomes [15, 44–46]. The Toronto group proposes that older donor age alone should not adversely impact outcomes, provided that all other indicators suggest excellent lung function [47]. Although we previously reported no significant difference in development of any PGD in a propensity-matched analysis between donors older and younger than 70 [15], our current multivariable analysis indicates that increasing donor age is associated with PGD grade 3 at 72 h and with 2-year all-cause mortality.

This retrospective study is hampered by several limitations. Although we adjusted for several potential confounding factors, the possibility of residual confounders cannot be excluded. Additionally, the time interval between the last reported PFR and the actual procurement procedure may vary between the Prague and Leuven cohorts, potentially introducing inter-center variability in the interpretation of donor lung function. Although donor blood gas assessment was performed using standardized ventilatory settings, this difference in timing may influence the interpretation of donor lung function. In the Prague cohort, PFR values are typically recorded immediately prior to donor transfer to the operating room, thus providing a more accurate reflection of the final physiological condition of the lungs. The Leuven cohort included a longer historical period and therefore contributed more mature follow-up data, whereas the Prague cohort contributed more recent cases with shorter follow-up. To reduce this limitation for the survival analysis, the primary survival endpoint was restricted to 2-year overall survival. Perioperative ECMO use also differed between centers, with Prague employing it routinely and Leuven using it selectively based on clinical indication. Immunosuppression was individualized in some cases and not strictly protocolized, which may introduce confounding. Markedly reduced PFR values were retained in the analysis as part of the continuous exposure range; however, their interpretation requires caution, as isolated low values may reflect transient atelectasis, reversible donor-management factors or extrapulmonary determinants of oxygenation rather than irreversible graft injury.

No formal adjustment for multiple comparisons was applied. This should be considered when interpreting the results, particularly because the study was retrospective and exploratory in nature and was designed to estimate clinically relevant associations rather than to test a single confirmatory hypothesis within a predefined family of comparisons. Although the principal clinical endpoints were prespecified, analyses of secondary endpoints and sensitivity models should be regarded as hypothesis-generating. Accordingly, p-values from secondary analyses should be interpreted descriptively, with emphasis placed on the direction, magnitude, confidence intervals, and consistency of effect estimates across models rather than on statistical significance alone.

In conclusion, donor PFR does not appear to be a significant risk factor for development of late PGD or for adversely affecting 2-year overall survival. However, it is associated with an increased risk of early PGD. Importantly, even when the donor PaO_2_/FiO_2_ ratio is low, surgical teams should still consider proceeding to evaluate the lungs *in situ*, as visual inspection, manual assessment, and intraoperative recruitment maneuvers may reveal acceptable graft quality. Further research is needed to delineate the distinctions between early and late PGD and to explore the broader implications of donor lung quality on post-transplant outcomes.

## Data Availability

The data analyzed in this study is subject to the following licenses/restrictions: the dataset contains sensitive patient-level information and is subject to institutional and GDPR regulations. Requests to access these datasets should be directed to RN, rene.novysedlak@lf1.cuni.cz or LJC, laurens.ceulemans@uzleuven.be.
