# Supplementary material for: Lower lung donor PaO2/FiO2 ratio correlates with early primary graft dysfunction, but does not impact overall survival after lung transplantation: a dual center study
Source: Transpl Int. 2026 Oct 9;39:16414. doi: 10.3389/ti.2026.16414 (PMC13690683; doi:10.3389/ti.2026.16414)
Supplement: Supplementary file 1 [file Supplementaryfile1.docx]

**Lower lung donor PaO_2_/FiO_2_ ratio correlates with early primary graft dysfunction, but does not impact overall survival after lung transplantation: a dual center study**

Supplementary Figure 1

**Figure S1.** Last PaO₂/FiO₂ ratio in accepted donors over time. Boxes indicate the interquartile range, horizontal lines the median, and whiskers the minimum and maximum values.
