# Supplementary material for: Lower lung donor PaO2/FiO2 ratio correlates with early primary graft dysfunction, but does not impact overall survival after lung transplantation: a dual center study
Source: Transpl Int. 2026 Oct 9;39:16414. doi: 10.3389/ti.2026.16414 (PMC13690683; doi:10.3389/ti.2026.16414)
Supplement: Supplementary file 2 [file Supplementaryfile2.docx]

**Lower lung donor PaO_2_/FiO_2_ ratio correlates with early primary graft dysfunction, but does not impact overall survival after lung transplantation: a dual center study**

Supplementary File 1 - Methods

**Institutional differences**

Perioperative ECMO use also differed between centers, with Prague employing it routinely and Leuven using it selectively based on clinical indication.

Immunosuppression was individualized in some cases and not strictly protocolized, which may introduce confounding.

Differences in post-transplant biopsy protocols exist between the two centers. In Leuven, surveillance is primarily conducted with endobronchial mucosal biopsies (EBMBs) at months 6, 9, 12, 18, and 24, while transbronchial biopsies (TBBs) are limited to postoperative days (POD) 30 and 90, or performed only if clinically indicated (e.g., unexplained functional decline or suspicious radiographic findings).

In contrast, Prague follows a protocol of routine TBBs at POD 30, 90, 180, and 360, resulting in approximately twice the number of tissue samples compared with Leuven. Additional methodological differences include the routine use of cryobiopsies and supplementary histological staining (e.g., CD20) in Prague, which are not yet standardized in Leuven. These factors may partially explain the higher reported incidence of acute cellular rejection (ACR) in Prague.
