# Supplementary material for: Lower lung donor PaO2/FiO2 ratio correlates with early primary graft dysfunction, but does not impact overall survival after lung transplantation: a dual center study
Source: Transpl Int. 2026 Oct 9;39:16414. doi: 10.3389/ti.2026.16414 (PMC13690683; doi:10.3389/ti.2026.16414)
Supplement: Supplementary file 3 [file Table1.docx]

**Lower lung donor PaO_2_/FiO_2_ ratio correlates with early primary graft dysfunction, but does not impact overall survival after lung transplantation: a dual center study**

Supplementary Tables

| **TABLE S1 Comparison of recipient characteristics and outcomes between Leuven and Prague** | | |
| --- | --- | --- |
|  |  |  |
| **Characteristics** | **Leuven (n=757)** | **Prague (n=227)** |
| ***Donor*** |  |  |
| *Type donor, n (%)* |  |  |
| DBD | 581 (76.74) | 222 (97.80) |
| DCD | 176 (23.25) | 5 (2.20) |
| Age at donation, years | 52 (41-62) | 46 (32-57) |
| *Sex, n (%)* |  |  |
| Male | 423 (55.88) | 118 (51.98) |
| Female | 334 (44.12) | 109 (48.02) |
| Body mass index, kg/m2 | 25 (22-27) | 24 (22-27) |
| Time on ventilator, days | 3 (2-6) | 4 (3-5) |
| Donor PaO_2_/FiO_2_, mmHg | 440 (384-500) | 420 (366-471) |
| ***Recipient*** |  |  |
| Age at transplant, years | 58 (50-62) | 56 (46-62) |
| *Sex, n (%)* |  |  |
| Male | 387 (51.13) | 144 (63.44) |
| Female | 370 (48.88) | 83 (36.57) |
| Body mass index, kg/m2 | 22 (19-26) | 26 (21-28) |
| *Indication for transplant, n (%)* |  |  |
| ILD | 190 (25.09) | 109 (48.02) |
| CF | 89 (11.76) | 17 (7.49) |
| COPD | 418 (55.22) | 74 (32.60) |
| PAH | 25 (3.30) | 22 (9.69) |
| Other | 35 (4.62) | 5 (2.20) |
| Pre-operative ECMO support | 24 (3.17) | 12 (5.28) |
| ***Surgical*** |  |  |
| EVLP, n (%) | 26 (3.44) | 0 (0) |
| EVLP duration, min | 250 (163.5-316) | 0 (0) |
| *Type of transplant, n (%)* |  |  |
| Double lung | 746 (98.55) | 227 (100) |
| Single lung | 11 (1.45) | 0 (0) |
| *Type of incision, n (%)* |  |  |
| Thoracotomy (bilateral and unilateral) | 679 (89.93) | 43 (18.94) |
| Clamshell | 76 (10.07) | 184 (81.06) |
| *Total ischemic time, min* |  |  |
| First lung | 220 (174-272) | 241 (200-280) |
| Second lung | 396 (331-465) | 350 (300-395) |
| Intra-operative ECLS | 109 (14.39) | 203 (89.42) |
| ***Outcomes*** |  |  |
| PGD 3 at 24 h or death | 103 (13.61) | 40 (17.62) |
| PGD 3 at 72 h or death | 93 (12.29) | 29 (12.78) |
| PGD 3 within 72 h or death | 222 (29.33) | 84 (37.00) |
| ICU length of stay | 6 (4-13) | 15 (11-28) |
| Hospital length of stay | 28 (23-39) | 26 (21-42) |
|  |  |  |
| Data are expressed as median (IQR) if not otherwise indicated. Percentages for incision type are based on available data.  Abbreviations: BMI – body mass index; CF – cystic fibrosis; CI – confidence interval; COPD – chronic obstructive pulmonary disease; DBD – donor after brain death; DCD – donor after circulatory death; ECLS – extra-corporeal life support; ECMO – extra-corporeal membrane oxygenation; EVLP – ex-vivo lung perfusion; ICU - intensive care unit; ILD – interstitial lung disease; PAH – primary arterial hypertension; PGD - primary graft dysfunction | | |

| **TABLE S2 Multivariable logistic regression for PGD grade 3 at 24 h - Backward Model Building** | | | |
| --- | --- | --- | --- |
|  |  |  |  |
|  |  | **Multivariable logistic regression** | |
| **Variables** |  | **OR (95% CI)** | **p value** |
| Time on ventilator, days |  | 0.939 (0.889-0.993) | **.0263** |
| **Donor PaO_2_/FiO_2_** |  | 0.997 (0.994-0.999) | **.0067** |
| Recipient age at transplant, years | |  | .1129 |
| *Linear term* |  | 1.063 (0.954-1.186) |  |
| *Quadratic term* |  | 0.999 (0.998-1.000) |  |
| Recipient BMI, kg/m² |  | 1.073 (1.019-1.129) | **.0075** |
| *Recipient indication for transplantation* |  |  | **<.0001** |
| ILD |  | 1.004 (0.384-2.621) |  |
| CF |  | 0.424 (0.136-1.317) |  |
| COPD |  | 0.616 (0.225-1.686) |  |
| PAH |  | 9.924 (3.382-29.12) |  |
| Other |  | # | . |
| Total ischemic time second lung, min | |  | **.0014** |
| *Linear term* |  | 0.991 (0.982-1.000) |  |
| *RCS term 1* |  | 1.065 (1.018-1.114) |  |
| *RCS term 2* |  | 0.850 (0.754-0.957) |  |
|  |  |  |  |
| # = reference category for calculation of the odds ratio. For continuous variables modeled linearly, the odds ratio reflects the change in odds per 1-unit increase. Recipient age was modeled using a quadratic function, and total ischemic time of the second lung was modeled using restricted cubic splines. A backward selection strategy was applied with p = 0.157 as the critical p-value for retention in the model. This threshold corresponds to model selection based on the Akaike Information Criterion (AIC). | | | |
| *Abbreviations: BMI - body mass index; CF - cystic fibrosis; CI - confidence interval; COPD - chronic obstructive pulmonary disease; ILD - interstitial lung disease; OR - odds ratio; PAH - primary arterial hypertension; RCS - restricted cubic spline* | | | |

| **TABLE S3 Multivariable logistic regression for PGD grade 3 at 72 h - Backward Model Building** | | | |
| --- | --- | --- | --- |
|  |  |  |  |
|  |  | **Multivariable logistic regression** | |
| **Variables** |  | **OR (95% CI)** | **p value** |
| Donor age, years |  | 1.022 (1.007-1.037) | **.0034** |
| Donor BMI, kg/m² |  | 0.916 (0.861-0.976) | **.0062** |
| **Donor PaO_2_/FiO_2_** |  | 0.999 (0.996-1.001) | .3342 |
| Recipient age at transplant, years | | 0.300 (0.118-0.763) | **.0115** |
| Recipient BMI, kg/m² |  | 1.079 (1.021-1.139) | **.0069** |
| *Recipient indication for transplantation* |  |  | **<.0001** |
| ILD |  | 1.832 (0.566-5.932) |  |
| CF |  | 1.027 (0.292-3.610) |  |
| COPD |  | 1.045 (0.310-3.524) |  |
| PAH |  | 12.27 (3.513-42.82) |  |
| Other |  | # |  |
| Total ischemic time second lung, min | | 1.002 (1.000-1.004) | **.0382** |
|  |  |  |  |
| # = reference category for calculation of the odds ratio. For continuous variables modeled linearly, the odds ratio reflects the change in odds per 1-unit increase. Recipient age was modeled using a log-transformed function at 72 h. A backward selection strategy was applied with p = 0.157 as the critical p-value for retention in the model. This threshold corresponds to model selection based on the Akaike Information Criterion (AIC). | | | |
| *Abbreviations: BMI - body mass index; CF - cystic fibrosis; CI - confidence interval; COPD - chronic obstructive pulmonary disease; ILD - interstitial lung disease; OR - odds ratio; PAH - primary arterial hypertension; RCS - restricted cubic spline* | | | |

| **TABLE S4 Univariable and multivariable Cox regression for CLAD-free survival** | | | |  |  |
| --- | --- | --- | --- | --- | --- |
|  |  |  |  |  |  |
|  | **Univariable Cox regression** | | **Multivariable Cox regression** | |  |
| **Variables** | **HR (95% CI)** | **p value** | **HR (95% CI)** | **p value** |  |
| ***Donor*** |  |  |  |  |  |
| *Type donor* |  |  |  |  |  |
| DCD | 0.816 (0.613-1.086) | .1627 | 0.761 (0.549-1.055) | .1015 |  |
| DBD | # | . | # | . |  |
| Donor age, years | 1.010 (1.003-1.017) | **.0077** | 1.009 (1.001-1.017) | **.0315** |  |
| *Donor sex* |  |  |  |  |  |
| Female | 0.957 (0.775-1.180) | .6782 | 0.808 (0.582-1.123) | .2042 |  |
| Male | # | . | # | . |  |
| Donor BMI, kg/m² | 0.996 (0.973-1.020) | .7655 | 0.982 (0.955-1.010) | .1972 |  |
| Time on Ventilator, days | 0.989 (0.965-1.013) | .3668 | 0.997 (0.970-1.024) | .8146 |  |
| **Donor PaO_2_/FiO_2_** | 0.999 (0.998-1.001) | .3155 | 0.999 (0.998-1.001) | .4567 |  |
| ***Recipient*** |  |  |  |  |  |
| Recipient age at transplant, years | 1.018 (1.008-1.027) | **.0002** | 1.003 (0.988-1.018) | **.6862** |  |
| *Recipient sex* |  |  |  |  |  |
| Female | 0.987 (0.801-1.216) | .9011 | 1.185 (0.852-1.649) | .3130 |  |
| *Male* | # | . | # | . |  |
| Recipient BMI, kg/m² | 1.036 (1.011-1.061) | **.0038** | 1.026 (0.998-1.055) | .0697 |  |
| *Recipient indication for transplantation* | |  |  |  |  |
| ILD | 1.594 (0.854-2.976) | **.0058** | 1.403 (0.734; 2.680) | .4833 |  |
| CF | 0.844 (0.424-1.679) | . | 1.011 (0.491; 2.081) |  |  |
| COPD | 1.529 (0.833-2.805) | . | 1.467 (0.764; 2.818) |  |  |
| PAH | 0.844 (0.350-2.037) | . | 0.864 (0.352; 2.120) |  |  |
| Other | # | . | # |  |  |
| *Recipient Incision Type* |  |  |  |  |  |
| Clamshell | 1.033 (0.787-1.355) | .8169 | 1.040 (0.770-1.404) | .7989 |  |
| Thoracotomy (bilateral and unilateral) | # | . | # | . |  |
| Total ischemic time second lung, min | 1.000 (0.999-1.001) | .6849 | 1.000 (0.999-1.002) | .6957 |  |
| *EVLP* |  |  |  |  |  |
| Yes | 0.758 (0.415-1.382) | .3656 | 0.798 (0.431-1.478) | .4739 |  |
| No | # | . | # | . |  |
|  |  |  |  |  |  |
| *# = reference category for calculation of the hazard ratio. For continuous variables, the hazard ratio reflects the change in hazard when the variable increases by 1 unit.*  *Abbreviations: HR - hazard ratio; CI - confidence interval; DCD - donor after circulatory death; DBD - donor after brain death; BMI - body mass index; ILD - interstitial lung disease; CF - cystic fibrosis; COPD - chronic obstructive pulmonary disease; PAH - primary arterial hypertension; EVLP - ex-vivo lung perfusion* | | | | |  |
|  |  |  |  |  |  |
|  |  |  |  |  |  |
